# Free versus purchased mosquito net ownership and use in Budondo sub-county, Uganda

**DOI:** 10.1186/s12936-018-2515-y

**Published:** 2018-10-16

**Authors:** Patricia Moscibrodzki, Molly Dobelle, Jessie Stone, Charles Kalumuna, Yueh-Hsiu Mathilda Chiu, Nils Hennig

**Affiliations:** 1 Icahn School of Medicine at Mount Sinai, 1 Gustave L. Levy Place, New York, NY 10029 USA; 2 Soft Power Health Uganda, P.O. Box 392, Kyabirwa, Uganda

**Keywords:** Malaria, Long-last insecticidal nets, Ownership, Use, Uganda, Households

## Abstract

**Background:**

While the distribution of mosquito bed nets is a widely adopted approach for malaria prevention, studies exploring how the usage of a net may be influenced by its source and other factors remain sparse.

**Methods:**

A standardized questionnaire and home-visit observations were used to collect data from 9 villages in Budondo sub-county, Uganda in 2016. Household- and individual-level data were collected, such as bed net ownership (at least one net versus none), acquirement source (free versus purchased), demographics, as well as knowledge of malaria and preventative measures. Net-level data, including alternative uses, and bed net quantity and integrity, were also collected. Mixed effects logistic regression models were performed to identify the key determinants of bed net use.

**Results:**

Overall, the proportion of households with at least one bed net was 40%, while bed net availability was only reported among 27% of all household members. Awareness of the benefits of bed net use was statistically significantly associated with ownership of at least one net (OR = 1.72, 95% CI 1.11–2.68, p = 0.02). Among those who own net(s), the odds of a bed net being correctly used (i.e., to sleep under) after adjusting for potential confounders were significantly lower for nets that were obtained free compared to nets that were purchased by the owners themselves (OR = 0.33, 95% CI 0.21–0.51, p < 0.01), resulting in an alternative use of the net. Other factors such as female gender, children ≤ 5 years old, and pregnancy status were also significantly associated with having a net to sleep under (all p < 0.01).

**Conclusion:**

Understanding inter- and intra-household net-use factors will help malaria control programmes more effectively direct their efforts to increase public health impact. Future studies may additionally consider socioeconomic status and track the lifetime of the net.

## Background

Malaria is endemic in the world and remains one of the most important diseases in Uganda, causing significant morbidity, mortality and negative socio-economic impact [1–3]. Of the 18 countries that account for 90% of *Plasmodium falciparum* infections in sub-Saharan Africa, Uganda ranks third in the total number of infections [1]. Hospital records collected in Uganda in 2014 suggested that malaria is responsible for approximately 30–50% of outpatient visits, 15–20% of admissions, and 9–14% of inpatient deaths [2]. Children under age 5 are at high risk because of low immunity against the disease [4]. In addition, pregnant women are also more susceptible to malaria infection due to hormonal and immunological changes, increasing their risk of illness, severe anaemia and death [5].

Long-lasting insecticidal nets (LLINs) are an important public health strategy for malaria prevention [6]. The ownership and use of LLINs has been shown in multiple settings across sub-Saharan Africa to reduce clinical episodes of malaria and all-cause child mortality [7–11]. The Uganda Malaria Reduction Strategic Plan (2014–2020) supports universal access to LLINs through mass campaigns and routine distribution channels [3]. Treating mosquito bed nets with insecticides provides dual protection: nets provide a direct barrier against host-biting mosquitoes for the person(s) sleeping under them, and mosquitoes may be killed if they come into contact with insecticides [10]. The toxicity and repellency induced by the pyrethroid insecticide-treated LLINs can have important community-wide effects on reduced vector density [7, 11–13]. Furthermore, LLINs have been shown to reduce the burden of malaria, especially among children ≤ 5 years old and pregnant women who are most vulnerable to malaria [9, 11].

A core focus of free mosquito bed net distribution campaigns has been equitable coverage within and between populations—with the goal of reducing disparities in access to preventive methods for economically or socially disadvantaged sub-populations. Previous studies show that large-scale, free net distribution campaigns can reduce inequities in household net ownership across socio-economic gradients [14, 15]. On the demand-side, much attention has been paid to the need for subsidization of the cost (or free) of bed nets as a means to increase bed net coverage, because cost has been identified as an important barrier to bed net ownership [16]. However, mosquito bed net ownership itself is not always synonymous with proper utilization [17]. Despite the overall accepted effectiveness of “universal coverage”, generally defined by 1 LLIN per 2 people, through free-distribution campaigns, there remain barriers to the use of bed nets in vulnerable households, which make rapid scale-up of bed net coverage difficult to achieve [16].

Without proper education, provision of free nets may not be enough to reach coverage for the required 80% of households as indicated by The World Health Organization (WHO) to provide community-wide “mass” protection from malaria [16]. Reports of misuse or alternate use of subsidized or free nets complicate the malaria prevention efforts of government’s and non-governmental organizations (NGO) seeking to promote bed net coverage especially among poorest individuals [16]. Studies show the rate of mosquito bed net use in net-owning households has been seen to be substantially less than 100% [18]. This indicates that ownership is not the only obstacle to achieving the reductions in malaria morbidity and mortality associated with bed net use, but also importantly, individuals who own mosquito bed nets must use them properly in order for the potential health impact to be fully realized [18].

Very few studies have investigated the status and patterns of net retention and continued use following free net distribution [10]. It has been postulated that, over the long-term, households that receive nets free of charge may experience greater net attrition compared to households that purchase nets. For example, in a study in Ghana, Senegal, Nigeria and Uganda, nets from a free distribution campaign were six-times more likely to be given away than nets purchased or obtained through other means [19]. Marginalized populations may be at a greater risk of net attrition following free-distribution, compared to less marginalized populations, if a lack of resources incentivizes an alternative use of the net [10]. To date, understanding of the retention of bed nets by households remains limited. Understanding patterns of bed net ownership and use can provide contextual insight into possible entry points for interventions, including those targeted at increasing rates of bed net access, hanging, and sleeping under.

Determining the context in a given population—lack of availability or failure to utilize available nets—is operationally important to inform a subsequent response. Thus, ‘targeted’ approaches to mosquito bed net promotion has been encapsulated in a recently proposed framework designed to inform “evidence-based and country-specific strategies to increase population coverage with [bed nets] and work towards the interruption of malaria transmission” [18]. Three categories of mosquito bed net non-use are recognized within this framework: (1) living in households with no nets present; (2) living in households owning, but not hanging a net; and (3) living in households owning and hanging, but not sleeping under a net [18]. Depending on which category is found to account for failing to use mosquito bed nets, the resulting interventions should focus on either improving net availability (category 1), encouraging the hanging of nets (category 2), or targeting individuals to encourage proper use of an existing net (category 3) [18].

This framework usefully highlights the variability for reasons of not using the mosquito bed nets and the importance of tailoring intervention strategies accordingly; however, the necessary interventions to promote greater mosquito bed net use among those who already own bed net(s) are likely to be more complex than, for example, those who do not own any bed nets. Various reasons may be attributed to not properly using the bed nets in net-owning households, including practical barriers of being unable to hang a net and social factors that render mosquito bed net use impractical or unimportant [20]. Interventions to increase actual use of bed nets have focused on subsidizing the costs associated with net ownership and providing education about malaria transmission and prevention. However, very limited malaria preventive interventions have focused on behavioural incentive tools, which have only recently been studied in developing countries to promote healthy behaviours and have proven to be effective in encouraging healthy behaviours, such as weight loss and tobacco cessation [16]. In recent years, performance-based incentives have been more widely applied to the promotion of behaviours related to health problems in developing countries [21], such as child immunizations [22], nutrition [23], maternal care [24], and tuberculosis detection and treatment [25]. These evidences suggest that, with sufficient statistics and planning, strategies incorporating incentives to increase bed nets use could potentially be more efficient than traditional distribution campaigns [16]. Therefore, it is important to understand and identify the crucial factors that affect proper bed nets use within the context of freely-obtained versus purchased nets.

To address these gaps, the status of net use components, namely, the correct net use of households with purchased versus freely received nets, households that use nets for alternative purposes, and the effect of perceived benefits of bed net-use on behaviour change were investigated. Specifically, the purpose of this analysis was to identify the important factors that are predictive of: (1) household bed net ownership, (2) correct use of bed nets, and (3) whether individuals within a household have bed nets to sleep under.

## Methods

### Study design and sample size

Jinja district, located approximately 80 km east of Kampala, is an area of perennial malaria transmission in eastern Uganda. The district has 11 subcounties that range from peri-urban to rural areas in the north, where malaria transmission is more intense. In this area of high endemicity, there are ongoing efforts underway to help achieve greater coverage of mosquito bed nets. These including free distribution in government health centers, selling nets with a reasonable price in the market, NGOs in the district offering free nets or nets with a price, and private sales of subsidized nets. Of note, the last preceding free mass mosquito net distribution campaign in Jinja district led by the government occurred in November 2013. Budondo sub-county of Jinja district was selected as the field site based on its geographical distance to Soft Power Mukagwa Allan Stone Community Health (SMACH) organization, which has been active for over 10 years in this area. According to the 2014 Uganda Census data, Budondo sub-county has a population of approximately 51.5 thousand people among 39 villages [26]. In 2014, malaria was the third most commonly treated condition at SMACH clinic despite efforts to reduce incidence and prevalence of malaria through aforementioned control methods. Further, periodical inspections revealed nets being used for many alternative purposes other than for sleeping under. Thus, a five-phase project was designed to examine the interplay of net sources with their correct use in 5 parishes within Budondo sub-county (i.e., 1 parish per phase/year): Ivanumba, Namizi, Kibibi, Nawangoma, and Buwagi, which were planned for 2015, 2016, 2017, 2018, and 2019, respectively. This present study focuses on the analyses using the data collected during the second phase, which was conducted in Namizi parish in 2016. Specifically, home visits and standardized questionnaires were conducted in 9 villages within the Namizi Parish (Buyala A, Buyala B, Buyala C, Buyala T/C, Kabowa Kampala, Kabowa T/C, Namizi Central, Namizi East, Namizi West) from June 2016 to August 2016. These villages are largely rural agricultural communities situated in close proximity to the Nile River.

All households within Namizi parish were eligible for this study. The household listing was shared by the Village Chiefs and Village Health Workers based on the population figures obtained from the 2014 Uganda Census, which consists of 2423 households across all nine villages. There was attempted contact with all houses on the household listing with no exception. During the study period, permissions to collect data from a total of 1815 households (74.9%) was obtained. Households that did not participate in the study (26.1%) were due to absence of any adults or legally capable individuals in the household during home visits. Prior to initiation of household assessments in each village, two research teams, each consisting of a trained graduate research investigator, a Soft Power Mukagwa Allan Stone Community Health (SMACH) translator, and a Village Health Worker (VHT), met with the respective village chairperson to discuss the survey and the study. After fully explaining the protocols and obtaining the approval from the chairperson, the research team began data collection in each village. The graduate research investigator was responsible for conducting the assessment with the assistance of the SMACH translator, while the VHT member served as a community representative to ensure more accurate and higher response rates to each question in the survey. The graduate research investigator and SMACH translator validated the survey questionnaires to ensure inter-rater reliability and to identify and verify translations. All study procedures were approved by the human studies committees at Mbarara University of Science and Technology and the Uganda National Council for Science and Technology. The participants provided written consents in their preferred language.

### Data collection

The number and composition of households in each village were verified by the respective village chairperson and the VHT prior to questionnaire administration. One questionnaire was administered to the head of the household or a stand-in member of the household, verified by the VHT. The questionnaire was verbally administered by the local SMACH translator, in the local language (Luganda or Lusoga). Participants were first asked how malaria was transmitted and what the benefits and barriers are to sleeping under a net. These were open-ended questions and all answers were recorded. They were then asked the total number of people, nets, and sleeping spaces in the household. Households that owned at least one net for sleeping were asked to provide the source (free or purchased) of each net, and whether the nets were used to sleep under. Free nets included nets that were given to the owner during the national free net distribution campaign or distributed via public/private clinics or NGOs. Purchased nets included any net the owner had purchased at either a subsidized or unsubsidized cost from a market, shop, NGO or health clinic such as Soft Power. The household was then asked if they owned any net(s) that was being used for an alternative purpose (i.e. used for any purposes other than to sleep under, such as fencing for a chicken coop), and if so, what it was being used for and if it was free or a purchased net. For households with a net used for sleeping, the research team then further inspected the net condition, material, and hanging status. Condition was recorded as excellent (< 2 holes of < 2 cm in diameter), fair (2 to 9 holes of < 2 cm in diameter), or poor (≥ 10 holes of < 2 cm, or ≥ 1 large hole of ≥ 2 cm). Only holes that were partially or completely visible after the net was tucked under the mattress were counted. Material (LLIN status) was determined upon inspection and recorded as polyethylene or polyester. Hanging status was recorded as hanging correctly, hanging incorrectly, or not hanging. Finally, participants were asked to provide the age, gender and pregnancy status of each person who had a net to sleep under, as well as of each person in the household who did not have a net to sleep under.

### Definitions of outcomes

- Household-level outcome: Bed net ownership; defined as a household owning at least one net intended for sleeping.
- Net-level outcome: Correct bed net use; defined as a bed net intended for sleeping, regardless of whether it was hanging or not hanging at the time of inspection (i.e., not used for an alternative purpose).
- Individual-level outcome: Having a net to sleep under; defined as a person having a net to sleep under regardless of whether they slept under the net alone or shared it with another individual (i.e., a person having access to bed net(s) for sleeping purpose).

### Exposures of interest

- Factors considered as potential predictors of the outcomes of interest described above include the age of the individual (children ≤ 5 years), whether the individual was pregnant, the source of the net (purchased or free), household-level perceived benefits and household knowledge of malaria.

### Statistical analysis

First, descriptive statistics of household-level, net-level and individual-level variables were derived. The proportion (%) of households owning at least one net from each household-level cross-sectional survey was calculated. The distributions of people with access to bed net(s) within their household and the number of nets owned were also calculated.

A series of univariable logistic regressions examining the associations between potential explanatory variables and three outcomes in separate models was performed (i.e., house-level, net-level, and individual level outcomes). Multivariable-adjusted logistic regression were then conducted, using a stepwise process with an inclusion criterion of p < 0.1 based on the explanatory variables corresponding to each outcome. Potential collinearity between predictor variables was also assessed. Given the possibility of violation of the assumption of model independence from repeated net measures on the same households, as well as possible household-level effects on the outcome, a random effects model with random intercepts to account for clustered effects was also performed. A likelihood ratio test comparing the mixed effects model to an ordinary logistic regression model was performed to determine whether there were improvements in the model fit.

The independent explanatory variables considered in the analysis on household-level outcome (bed net ownership) included: number of people in the household, at least one child ≤ 5 years, at least one woman pregnant, knowledge of where malaria comes from, and the perceived benefits of using a bed net. For the analysis on net-level outcome (whether a net was used to sleep under), number of bed nets within the household, number of residents per household, number of children ≤ 5 years in household, number of pregnant women in household, household-perceived benefit of mosquito bed net, household-knowledge of where malaria comes from, and source of net obtainment were considered. Finally, for the analysis on individual-level outcome (whether an individual had access to bed net to sleep under), gender, pregnancy status, age ≤ 5 years old, total number of residents living in the same household, number of nets in the household, number of sleeping spaces in the household, whether the individual lived in a household that used a net for an alternative purpose, and the household’s perceived benefits and knowledge of malaria were considered.

Results from likelihood ratio tests comparing the mixed effects model to an ordinary logistic regression model was insignificant for both household-level and net-level models. Thus, results from the multivariable logistic regression models were reported as the final model for these outcomes, adjusting for household-level effects through cluster robust standard errors, which ensured adjustment for household inter-cluster correlation. On the other hand, the results from likelihood radio tests for individual-level outcome revealed significant improvement in the model fit using mixed model, and thus the results from random mixed effects logistic regression with random intercepts to account for household effects were reported. Data analysis was performed using STATA version 14.2 software.

## Results

### Household characteristics

The survey questionnaire captured 8011 individuals in 1815 households among nine villages. Median household size was 4.4 persons with roughly 59% of households having at least one child 5 years or younger. Of the 8011 individuals, information on sleeping bed net was available from 7363 individuals. The mean age was 20.9 ± 18.5 years; 3999 (54%) were female and 3363 (46%) were male (Table 1). Children ≤ 5 years old and pregnant women accounted for 23.5% and 2.4%, respectively.

**Table 1:** Characteristics of study participants (7363 individuals from 1815 households) from nine villages in Budondo sub-county, Uganda

Variables	Frequency, n (%)
Age [mean (SD)]	20.9 (18.5)
Gender	
Female	3999 (54.3)
Male	3363 (45.7)
Pregnant	184 (2.4)
Child ≤ 5 years of age	1879 (23.5)
Household member size [mean (SD)]	4.4 (2.6)
Knowledge of malaria: where does malaria come from?	
No answer	379 (20.87)
Mosquitoes	1354 (74.6)
Contaminated water	16 (0.88)
Dust	14 (0.77)
Cold	8 (0.44)
Dirty environment, poor hygiene	15 (0.83)
Other	29 (1.60)
Perceived benefits of bed net	
No opinion	251 (13.8)
Less mosquito bites, less malaria	1524 (84.0)
Warmth, more comfortable	27 (1.5)
Less other insect bites	6 (0.3)
Other	7 (0.4)
Perceived barriers to use of bed net	
No barriers	575 (31.7)
Cost	915 (50.4)
Access	42 (2.3)
Too warm, less comfortable	98 (5.4)
Constricting, little space	1 (0.1)
Waiting for governmental net distribution	111 (6.1)
Other	73 (4.0)
Number of sleeping spaces	
1	424 (23.4)
2	534 (29.4)
3	419 (23.1)
4	196 (10.8)
5	97 (5.3)
6	65 (3.6)
≥ 7	80 (4.4)
Individual net use	
Individuals having no net to sleep under	5335 (72.5)
Individuals have a net to sleep under, but it was not hanging during home inspection	742 (10.0)
Individuals have a net to sleep under, and it was hanging during home inspection	1286 (17.5)

### Household-knowledge of malaria and mosquito net prevention

Household respondents were asked about the cause of malaria, and the benefits and barriers of sleeping under a mosquito net (Table 1). The knowledge that malaria is caused by a mosquito was fairly high (74.6%). A total of 86% of the households acknowledged that the bed nets can provide some type of benefits, whereas 84% answered that bed nets can protect oneself against malaria. A total of 68% households reported a barrier to use a bed net, and 50% reported that barrier being cost, whereas 32% of the total population surveyed reported no barriers.

### Bed net ownership, usage and condition

Among the 1815 households being visited/surveyed, only 734 (40%) households had at least one bed net used for sleeping purposes. The average number of self-reported bed nets used for sleeping per household was 1.75 (median = 1), and only 43.6% reported that at least one member of the household have been sleeping under a net. At the individual-level, 5335 (72.5%) individuals did not have a net to sleep under (20.3% children ≤ 5 years of age, 54% female, and 2.3% were pregnant), of which 4159 (56.5%) lived in a household with no nets. Of the 1083 children ≤ 5 years that do not sleep under a net, 79% lived in a household with no nets. Similarly, of the 122 pregnant women who did not sleep under a net, 86% lived in a household that did not own a bed net. Among the 2028 (27.5%) individuals who reported that they have access to a bed net to sleep under, 742 of their nets were not hanging during home inspections (Table 1). The majority of the reasons for not hanging the nets during the day time included “the net being washed” and “only hang the net at night”. Of note, 99.1% of the nets were used by ≤ 3 individuals at a time (including children), and 86.5% of the nets were used by ≤ 2 individuals at a time.

Among the 894 nets that were inspected (i.e., do not include those nets that were self-reported by the participants but were not seen by the interviewers) for net integrity and condition type, 64.8% were determined good (< 2 holes of < 2 cm), 18.5% were fair (2 to 9 holes of < 2 cm) and 16.7% were poor (≥ 10 holes of < 2 cm or ≥ 1 large hole of ≥ 2 cm) (Table 2). In addition, 87.6% of nets being inspected were hanging correctly (Table 2).

**Table 2:** Source, ownership and use of 1573 nets reported by 978 households with at least one net, including those used for an alternative purpose

Variable	Frequency, n (%)
Bed net source	
Soft Power Health Clinic	115 (7.3)
Government	1214 (77.2)
Shop/market	206 (13.1)
Hospital/clinic	23 (1.5)
NGO	11 (0.7)
Other	4 (0.3)
Purpose of net use	
For sleeping under	1257 (79.9)
Alternative use (i.e., not used for sleeping)	316 (20.1)
Material (LLIN status)	
Polyethylene	566 (36.0)
Polyester	327 (20.8)
Undetermined	680 (43.2)
Net condition among nets inspected (n = 894)	
Good (< 2 holes of < 2 cm)	579 (64.8)
Fair (2 to 9 holes of < 2 cm)	165 (18.5)
Poor (≥ 10 holes of < 2 cm or ≥ 1 large hole of ≥ 2 cm)	149 (16.7)
Hanging status among nets inspected (n = 894)	
Number of nets hanging correctly	783 (87.6)
Number of nets hanging but not correctly	7 (0.8)
Number of nets not hanging	104 (11.6)

At the net-level, a total of 316 (20%) nets were reported to be used for a range of alternative purposes (Table 2). Table 3 demonstrates in detail about the categories and frequency of alternative net use. In addition, strikingly, among all these nets used for alternative purposes, 92% were obtained for free.

**Table 3:** Alternative net use by category

Alternative net use	Frequency, n (%)
Gardening, plant protection, fencing	55 (16.0)
Chicken house	39 (11.3)
Bedding (sleeping mat, mattress support, bed sheets etc.)	100 (29.1)
Rope, clothesline	54 (15.7)
Sitting, chair cushion	13 (3.8)
Sponge, scrubber	31 (9.0)
Food storage, vegetable drying	8 (2.3)
Window screens, wall, curtain, room divider etc.	28 (8.1)
Other (garbage cover, toy, table cloth)	16 (4.7)

Based on n = 316 nets reported for alternative uses across 1815 households

### Determinants of owning a net, correct bed net use, and having a net to sleep under

#### Household-level

Results of the analyses at the household-level are shown in Table 4. The multivariable analysis demonstrated that households are significantly more likely to own at least one bed net if household members acknowledged any benefit of using bed nets (OR = 1.72 (1.11–2.68), p = 0.02). Other variables that were marginally significantly associated with owning at least one bed net in the household include: having at least one child ≤ 5 years (OR = 1.11 (0.99–1.25), p = 0.07) and the number of individuals within a household (OR = 1.05 (1.00–1.20), p = 0.05; corresponding to per person increase).

**Table 4:** Univariable and multivariable logistic regression analysis of determinants of owning at least one bed net among n = 1861 households

Variable	Univariable OR (95% CI), p-value	Multivariable OR (95% CI), p-value
Benefits		
No perceived benefits of using bed net	REF	REF
Any perceived benefit of using bed net	1.96 (1.27–3.03), < 0.01	1.72 (1.11–2.68), 0.02
Knowledge of malaria		
No knowledge, or belief malaria is caused by several varying factors	REF	–
Knowledge that mosquitoes are the sufficient and necessary cause of malaria	0.93 (0.73–1.18), 0.55	–
Children in household ≤ 5 years of age		
No children ≤ 5 years	REF	–
At least one child ≤ 5 years	1.21 (1.11–1.32), < 0.01	1.11 (0.99–1.25), 0.07
Pregnancy status		
Not pregnant	REF	–
Pregnant	1.11 (0.83–1.50), 0.49	–
Number of individuals per sleeping space (continuous)	1.22 (1.07–1.38), < 0.01	1.03 (0.88–1.20), 0.72
Number of people in household (continuous)	1.09 (1.05–1.13), < 0.01	1.05 (1.00–1.20), 0.05

#### Net-level

The multivariable analysis for total nets reported (N = 1573; including those used for alternative purpose), revealed that a net being obtained from free sources was associated with significantly decreased odds of this net being used for the correct purpose (i.e. using a net for sleeping under rather than for an alternative use) (OR = 0.33 (0.21–0.51), p < 0.01) (Table 5). Other variables that are statistically significantly predictive of a net being correctly used include: number of nets within the household (OR = 5.65 (3.98–8.08), p < 0.01; corresponding to per one net increase) and having at least one pregnant women in the household (OR = 1.83 (1.19–2.83), p < 0.01) (Table 5).

**Table 5:** Univariable and multivariable logistic regression analysis of determinants of a net being used correctly (N = 1573 nets)

Variable	Univariable OR (95% CI), p-value	Multivariable OR (95% CI), p-value
Source of nets		
Purchased	REF	REF
Free	0.28 (0.18–0.43), < 0.01	0.33 (0.21–0.51), < 0.01
Benefits		
No perceived benefits of using bed net	REF	REF
Any perceived benefit of using bed net	2.06 (1.34–3.18), < 0.01	0.93 (0.66–1.33), 0.70
Knowledge of malaria		
No knowledge, or belief malaria is caused by several varying factors	REF	–
Knowledge that mosquitoes are the sufficient and necessary cause of malaria	1.20 (0.90–1.61), 0.21	–
Pregnant women in household		
Not pregnant	REF	REF
Pregnant	1.75 (1.08–2.81), 0.02	1.83 (1.19–2.83), < 0.01
Children in household ≤ 5 years (continuous)	1.10 (0.98–1.23), 0.09	0.95 (0.85–1.07), 0.39
Number of people in household (continuous)	1.04 (0.99–1.09), 0.15	–
Number of nets in household (continuous)	5.61 (4.54–6.93), < 0.01	5.65 (3.98–8.08), < 0.01

#### Individual-level

Table 6 demonstrates that results of the analyses in individual-level data (n = 7363 without any missing data on exploratory variables listed in the “Methods” section). In the univariable analysis, the factors significantly associated with an individual having a bed net to sleep under were children ≤ 5 years (OR = 1.36 (1.09–1.70), p < 0.01), pregnant women (OR = 2.64 (1.49–4.66), p < 0.01), living in a household with any perceived benefits of net-use (OR = 4.66 (1.48–14.59), p < 0.01), and number of nets owned by the household that the individual lives in (OR = 19.49 (15.18–25.03), p < 0.01; corresponding to per one net increase). Whereas, being male (OR = 0.78 (0.65–0.93), p < 0.01), and the number of individuals living in the same household (OR = 0.43 (0.84–1.01), p = 0.07; corresponding to per one person increase), was associated with decreased likelihood of having a bed net to sleep under.

**Table 6:** Univariable and multivariable random mixed effects logistic regression analysis of determinants of having access to bed net to sleep under among 7363 individuals

Variable	Univariable OR (95% CI), p-value	Multivariable OR (95% CI), p-value
Gender		
Female	REF	REF
Male	0.78 (0.65–0.93), < 0.01	0.85 (0.71–1.02), 0.09
Age		
> 5 years	REF	REF
≤ 5 years	1.36 (1.09–1.70), < 0.01	1.75 (1.40–2.18), < 0.01
Pregnancy status		
Not pregnant	REF	REF
Pregnant	2.64 (1.49–4.66), < 0.01	2.66 (1.58–4.53), < 0.01
Number of nets in household (continuous)	19.49 (15.18–25.03), < 0.01	17.64 (14.15–21.98), < 0.01
Number of people in household (continuous)	0.43 (0.84–1.01), 0.07	0.59 (0.56–0.63), < 0.01
Alternative use		
Living in household that does not use nets for alternative purposes	REF	–
Living in household that uses nets for alternative purposes	0.59 (0.30–1.14), 0.12	–
Benefits		
No perceived benefits of using bed net	REF	–
Any perceived benefit of using bed net	4.66 (1.48–14.59), < 0.01	1.88 (1.06–3.32), < 0.01
Knowledge of malaria		
No knowledge, or belief malaria is caused by several varying factors	REF	–
Knowledge that mosquitoes are the sufficient and necessary cause of malaria	0.63 (0.34–1.13), 0.13	–

Results from the multivariable analysis were similar to the results from the univariable analyses. It again showed that living in a household that acknowledges any benefits of bed nets was significantly associated with higher odds of having a net to sleep under (OR = 1.88 (1.06–3.32), p < 0.01). Furthermore, being ≤ 5 years (OR = 1.75 (1.40–2.18), p < 0.01), or being pregnant (OR = 2.66 (1.58–4.53), p < 0.01) was significantly associated with having a net to sleep under.

## Discussion

This is one of few studies to date that attempted to elucidate the factors influencing bed nets usage status (e.g., bed nets ownership, adequacy and purpose of bed nets use) in Uganda that may inform future intervention strategies. Notably, freely-obtained bed nets (e.g., obtained via national free net distribution campaign, or distributed via public and private clinics or NGOs) were significantly more likely to be misused or used as an alternative purpose rather than to sleep under. As anticipated, lack of awareness of any benefits that can be provided by bed nets was associated with inadequate use of bed nets and decreased bed nets ownership in this study population. However, interestingly, having knowledge that mosquitoes are vectors of malaria was not predictive of increased ownership or decreased inadequate use of bed nets.

This study also demonstrated that only 40% of the households had at least one net among the 1815 households visited in Uganda; and only 27% individuals among the 8011 included in this study correctly have bed nets available to them for sleeping purpose. Despite the relatively low household bed net ownership compared to previously reported high coverage rates elsewhere including Sierra Leone (87.6%), Togo (96.7%) and Ethiopia (91.0%) [27–29], the results from this present study in Budondo subcounty in Uganda showed a similar pattern of lower bed net use than bed net coverage as in those other countries. Relatedly, coverage in these three other settings varied from 65.0% in Ethiopia to 68.3% in Togo and 76.5% in Sierra Leone [12]. Interestingly, household ownership of at least one net in the Ministry of Uganda Malaria Indicator survey (2014) were reported to be 84.6% for East Central region which encompasses the area covered within this paper [1]. This may suggest a global discrepancy of reported rates.

The consistent gap between household possession of bed nets and individual correct usage draws attention to an important aspect of human behaviour [30]. This study found that neither the complementary distribution of insecticidal nets nor the knowledge on malaria transmission automatically translated into correct usage. Therefore, it may be inferred that information disseminated needs to be culturally-sensitive and based on existing positive beliefs and behaviour if it is to be acceptable by the community.

The finding in this study that having a perceived benefit of net-use was significantly associated with a higher likelihood of the household owning at least one net, as well as individuals having a net to sleep under, is particularly important given its influence on aspects of preventative measures against malaria [31]. The interesting disconnect between the knowledge that mosquitoes propagate malaria and the corresponding benefit of using mosquito nets to prevent malaria, was showcased through the analyses at all levels; the household-, net- and individual-levels. The household-representative’s knowledge that malaria comes from mosquitoes was not statistically significant with households owning a net, nets being used correctly, or individuals sleeping under a net. The perceived benefits provided by respondents in the study were largely stated to be protection from malaria, but non-health benefits were also valued in this study. Other studies have also highlighted the non-malaria benefits in using nets as perceived by communities. For example, a study in Gambia showed that privacy obtained by sleeping under a net to be a motivating factor [32], and in Zanzibar, aspects of comfort appeared to play a key role in personal decisions [33]. The importance of emphasizing the non-health advantages to sleeping under nets have also been suggested in response to seasonal fluctuations in mosquito numbers which can affect the perceived threat of malaria and, by association, net use [8]. This evidence suggests future educational campaigns could benefit from advocating non-malaria benefits of net use, in addition to malaria-prevention, to provide a long-term rationale for consistent use.

This study also suggests that being a child ≤ 5 years was significantly associated with sleeping under a net, and noteworthy, also associated with the household owning a net. These promising results indicate that those most vulnerable to malaria are being protected [4]. Interestingly, among children ≤ 5 years not sleeping under a net, 79% lived in a household that did not own a bed net. This result, that the largest category of non-use is directly related to household bed net ownership, indicates that accessibility is still a barrier to bed net use for this population. On the other hand, children ≤ 5 years of age living in households owning a bed net made up a relatively small proportion (21%) of not sleeping under a net, which suggests that caretakers recognize the importance of protecting young children with bed nets.

Similarly, being pregnant/having pregnant women in household was significantly associated with sleeping under a net and increased odds a net is used correctly. Of the 122 pregnant women who did not sleep under a net, 86% lived in a household that did not own a bed net. This also suggests that households owning bed nets recognize the importance of protecting pregnant women with bed nets. Use of bed nets among pregnant women is associated with lower prevalence of malaria infection, fewer premature births and significant reductions in all-cause maternal anaemia [29]. Given the priorities for children ≤ 5 years of age and for women of reproductive age, the popular belief that the man of the household often takes the net for his own use has been rejected in this study and others [34].

However, bed net ownership will have little impact on relieving the burden of malaria unless people sleep under bed nets. Many large-scale programmes have encountered challenges in consistent use of bed nets [29]. Once households acquire a bed net, there are still other considerable factors that determine their actual use [16]. Reports of misuse of subsidized nets complicate the efforts of government and non-governmental organization (NGO) malaria prevention programmes seeking to promote bed net coverage and use in poor countries [16]. This study found that households who obtained nets for free had lower odds of using them correctly. Comparably, there are documented cases of nets being used for other purposes in Ethiopia [29], for drying fish near Lake Victoria [35], and for fishing in Lake Tanganyika [36]. A multi-country analysis across four countries found that campaign nets (obtained free) were nearly six times more likely to be given away than non-campaign nets [33].

Another important net-use issue is that of nets that go unused at all. One study that investigated intra-household net use in Ethiopia, Ghana, Mali, Nigeria, Senegal and Zambia noted that nets that were paid for were used more than free nets [34]. Another study showed that charging for nets was associated with a high level of usage among the poorest quintile (up to 50%), though with aggressive social marketing of the nets [37]. One proposed explanation has been that of economic theory—a negative price (i.e. paying consumer to use an item in the form of an incentive; free) may provide a negative quality signal and potentially devalue the item in the view of some consumers [16]. As shown in a study in Mozambique where demographic variables were adjusted in the analyses, it was found that compared to non-pregnant women, pregnant women were 27 times more likely to sleep under a mosquito bed net if all bed nets were purchased, and 13 times more likely if all bed nets were received as a donation [38]. Given that Mozambique’s population is considered multi-dimensionally poor, the finding that more bed nets were purchased than received through donations, highlights that Mozambican strategies to achieve universal coverage through mass donation strategies have not yet been reached saturation—in order to maximize household mosquito bed net ownership and correct usage, a combination of both free distribution strategies as well as commercial options will likely be needed [38]. A mix of target subsidy programmes that combine discount vouchers and free distribution showed promising results in Nigeria, Senegal, Uganda and Zambia [29]. The tremendous gains achieved in all four countries suggested that free distribution to poorer families should complement rather than replace giving those with more resources the option to buy a net and will result in optimal ownership [39].

The recently proposed framework that designates households of bed net non-use (no net, having net but not hanging, and hanging but not slept under), could similarly be applied at the individual-level. The findings of this study suggest that 72.5% of individuals lacked access to a sleeping net, and 9.3% had a net in their household but it was not hanging. The third category was unattained due to a lack of individual data on bed net use the previous night. Identifying specific areas of non-use may enable malaria programmes to make informed decisions about which intervention strategies may improve bed net use. Outreach, informed by non-use data, could be aimed at (1) improving bed net access, (2) encouraging hanging bed nets and (3) targeting individuals to sleep under existing bed nets [40].

While this study provides informative and significant results with analyses in a reasonably large sample size, some caveats must also be acknowledged. First, the cross-sectional nature of this study is able to show associations but do not imply causal relationships; thus, future longitudinal or prospective studies are needed to elucidate causal pathways for bed net use and non-use as well as sustainability. Second, households undertook the interview but refused research team’s spot-observations of nets in the home may introduce recall bias. However, it is likely to be non-differential misclassification as the participants were not informed about the purpose of the study and thus misclassification of the outcome is less likely to be dependent on exposure of interest. On the other hand, the findings from this study may not be generalizable to the population that usually do not have any adults in the household during the day time (i.e., typical time of home visits by research team). For example, households with multiple individuals working outside the home may be more likely to be educated or wealthier and thus more likely to use nets. Third, some of the factors investigated may be an indicator for poverty in the study population and hence, may be confounded by socio-economic variables that were not available in this study. Fourth, accurate information on the age of the net was not obtained, which could be another explanatory factor, in addition to source of obtainment of the net, that is related to decreased correct usage of net due to wear and tear of the nets and being used for alternative purposes. Yet, given that literature suggested that the estimated useful life of nets are about 3–4 years [41], and given that the last preceding governmental free mass mosquito net distribution campaign in Jinja district occurred about 2.5 years before data collection of this study, the lifetime of the nets at time of study is not likely to hinder the ownership or condition of nets significantly. Future studies including an analysis of the age of nets would be advantageous to determine the likelihood of confounding by age of the net on the relationship between source of net obtainment and correct net use. Finally, missing values (~ 8%) of potential predictors in the individual-level data slightly reduced the sample size of the analyses for examining the predictors of whether an individual has a net to sleep under. Nonetheless, the distributions of basic characteristics such as gender and age were similar in those included and excluded in the analyses (p > 0.05).

## Conclusion

This study demonstrates that bed net use is associated with a number of factors related to household prevention of malaria such as the appreciation and awareness of a range of benefits, including malaria-related and non-malaria benefits, arising from bed net use, as well as the potentially adverse effects of nets obtained free, which should be taken into consideration and incorporated into programme policy. The results of this study support the data from some previous studies suggesting that bed nets that were paid for were more likely to be used and used adequately than those obtained free. Thus, a segmentation strategy targeting free bed nets to rural and poorest households combined with support for the commercial sector in urban and better-off areas may optimize bed net coverage as well as promote bed net use. Furthermore, data on specific non-use patterns that depend on the cultural beliefs or factors that drive the behavioural decisions of the target population may inform cost-effective intervention strategies to improve bed net coverage. These tailored strategies focused on benefit-based behavioural change may be likely to raise levels of use.
